# Impact of Prolonged Surgical Waiting Time on 5‐Year Progression‐Free Survival in Patients With Endometrial Cancer

**DOI:** 10.1111/jog.70175

**Published:** 2026-01-08

**Authors:** Ascharavadee Pulsawat, Sitchuphong Noothong, Nathapol Sirimusika

**Affiliations:** ^1^ Department of Obstetrics and Gynecology, Division of Gynecologic Oncology Hatyai Hospital Songkhla Thailand

**Keywords:** endometrial neoplasms, neoplasm recurrence, progression‐free survival, time‐to‐treatment

## Abstract

**Aim:**

To evaluate the impact of surgical waiting time (SWT) on 5‐year progression‐free survival (PFS). Secondary objectives were to evaluate 5‐year overall survival (OS) and prognostic factors for recurrence and OS in endometrial cancer (EC), considering Thailand's Key Performance Indicator (KPI) recommending surgery within 4 weeks.

**Methods:**

This retrospective cohort study included 377 patients with histologically confirmed EC who underwent primary surgery at Hatyai Hospital between October 2016 and September 2024. SWT was defined as the time from diagnostic biopsy to definitive surgery and categorized as early (≤ 4 weeks) or delayed (> 4 weeks). Survival outcomes were assessed using Kaplan–Meier curves and log‐rank tests. Prognostic factors were analyzed using Cox proportional hazards models.

**Results:**

The median SWT was 32 days. Median follow‐up was 23 months. Delayed surgery correlated with higher BMI, larger tumors, and increased recurrence (12.7% vs. 4.9%, *p* = 0.010). Advanced FIGO stage, non‐endometrioid histology, grade 3 tumors, LVSI, and delayed SWT were significant recurrence risk factors. Multivariate analysis confirmed advanced stage (HR: 5.15, *p* < 0.001) and SWT > 4 weeks (HR: 3.22, *p* = 0.011) as independent predictors. Recurrence risk increased with longer delays (> 6 weeks: HR: 3.22; > 8 weeks: HR: 3.16). Kaplan–Meier curves showed significantly reduced PFS with prolonged SWT, while its effect on 5‐year OS was not significant (*p* = 0.1).

**Conclusions:**

Surgical delays beyond 4 weeks were associated with reduced PFS and increased recurrence, supporting Thailand's KPI and underscores the importance of timely surgery. The short follow‐up may limit interpretation of long‐term outcomes. Longer follow‐up is warranted to confirm these results.

## 1 Introduction

Endometrial cancer (EC) is the most common gynecologic malignancy in developed countries, with a global age‐standardized incidence rate of 8.4 per 100 000 woman‐years [1]. Although EC has historically been less prevalent in South‐Central Asia, recent epidemiological data from Songkhla province in southern Thailand show a concerning upward trend, with incidence rising from 1.5 to 5.3 per 100 000 woman‐years in 2016 and projected to reach 8 per 100 000 by 2030 [2]. This increasing burden highlights the need for timely and effective treatment strategies in the region.

Surgical management, typically comprising total hysterectomy with bilateral salpingo‐oophorectomy and lymph node assessment, remains the cornerstone of treatment for early‐stage EC and is associated with favorable outcomes [3]. In contrast, patients with advanced disease often require adjuvant radiotherapy or chemotherapy to improve survival [3]. Among the various factors influencing prognosis, the timeliness of surgery has emerged as a critical determinant of outcomes in cancer care. Delays in treatment exceeding 6 weeks have been shown to significantly reduce survival in several malignancies, including breast, colorectal, lung, renal, and pancreatic cancers [4]. Similarly, in gynecologic malignancies such as early‐stage cervical and uterine cancers, delays beyond 8–12 weeks are associated with poorer progression‐free and overall survival [5, 6, 7].

The biological behavior of EC suggests that surgical delays may promote tumor progression through deeper myometrial invasion, lymphovascular space involvement, and earlier metastatic dissemination—factors known to be associated with recurrence and reduced progression‐free survival (PFS) [8, 9]. Despite this understanding, there is no clear consensus on the optimal interval between diagnosis and definitive surgery for EC. Most existing guidelines emphasize timely referral but lack specific, evidence‐based recommendations for the ideal timing of surgery.

In Thailand, the national Key Performance Indicator (KPI) mandates that surgery for cancer patients be performed within 4 weeks of diagnosis. However, this benchmark has not been substantiated by localized clinical outcome data, particularly in the context of endometrial cancer. The absence of such evidence leaves a critical gap in current cancer care policy and raises concerns regarding the appropriateness and applicability of existing timelines.

To address this gap, the present study aims to evaluate the impact of surgical waiting time (SWT) on 5‐year progression‐free survival (PFS) in patients with EC. Secondary objectives were to assess its effect on 5‐year overall survival (OS) and identify prognostic factors for recurrence and OS. These findings aim to guide clinical decision‐making and inform evidence‐based policies to optimize care for patients with EC in Thailand and similar healthcare systems.

## 2 Methods

**FIGURE 1:**
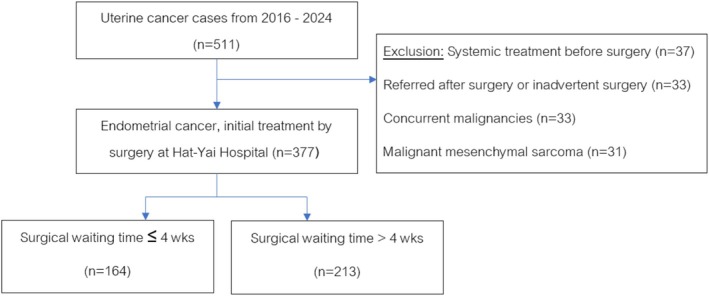
Study population flow diagram. A total of 511 uterine cancer cases diagnosed between 2016 and 2024 were identified. After applying exclusion criteria, 377 patients with endometrial cancer who underwent primary surgical treatment at Hat‐Yai Hospital were included. These were stratified into two groups according to surgical waiting time (SWT): ≤ 4 weeks (*n* = 164) and > 4 weeks (*n* = 213).

We obtained approval from the Ethics Committee of Hatyai Hospital (Study code: HYH EC 044‐68‐01) before the study. This retrospective cohort study reviewed data from patients diagnosed with EC between October 2016 and September 2024 at the Department of Obstetrics and Gynecology, Hatyai Hospital, a tertiary referral center in Southern Thailand. Eligible patients were those with a histopathological diagnosis of EC, classified under ICD‐10 codes C54 (malignant neoplasm of the corpus uteri) or C55 (malignant neoplasm of the uterus, unspecified), who underwent definitive surgery (Total abdominal hysterectomy and bilateral salpingo‐oophorectomy and/or lymph nodes dissection and/or peritoneal washing) at Hatyai Hospital. A total of 511 patients were identified. Patients were excluded if they received systemic chemotherapy prior to surgery, underwent inadvertent surgery, had malignant mesenchymal tumors (e.g., uterine sarcoma), or had concurrent malignancies. After exclusions, 377 patients were included in the final analysis (Figure 1).

Descriptive characteristics of EC, including age, menopausal status, body mass index (BMI), underlying diseases, parity, and tumor features such as the 2009 International Federation of Gynecology and Obstetrics (FIGO) stage, histological type (endometrioid, serous, mucinous, clear cell carcinoma, or carcinosarcoma), tumor grade, tumor size, lymphovascular space invasion (LVSI), recurrence status and sites of recurrence were collected. The final diagnosis of EC cases was based on histopathology reports. Postoperative adjuvant treatment (radiation and/or chemotherapy) was reviewed and documented, and the time to recurrence was recorded. Adjuvant therapy after surgery was administered according to surgical risk factors. Follow‐up after treatment was every 3 months in the first year, every 4 months in the second year, every 6 months in the 3rd to 5th years, and annually thereafter.

The SWT was calculated from the date of diagnosis (endometrial biopsy or curettage) to the date of definitive primary surgery. The primary outcome was the impact of SWT on 5‐year PFS, calculated from the date of surgery to the date of confirmed tumor recurrence. SWT was categorized as early (≤ 4, ≤ 6, and ≤ 8 weeks) or delayed (> 4, > 6, and > 8 weeks) for group comparisons. Patients who were lost to follow‐up or had no recorded recurrence during follow‐up were considered censored at the last known date when their status was available. The OS was measured from the date of surgery to the date of death or last follow‐up. Secondary objectives were to assess its effect on 5‐year OS and identify prognostic factors for recurrence and OS.

Descriptive data analysis was used to calculate frequencies for categorical data and means with standard deviations for quantitative data. All confidence intervals (CI) for parameters to be assessed were calculated with a significance level of alpha = 0.05, with a threshold for significance set at *p* < 0.05. Prognostic factors influencing PFS were presented as hazard ratios (HR) and 95% CI for each individual variable. Kaplan–Meier survival analysis was used to calculate the 5‐year PFS and 5‐year OS rates. The Log‐rank test was employed to compare the groups of interest. Cox proportional hazards regression analysis was performed to identify predictive factors associated with survival. Univariate analysis was conducted for each variable, and statistically significant variables were subsequently entered into the multivariate analysis. Statistical analysis was performed using R software (version 4.4.1).

## 3 Results

A total of 377 patients with endometrial cancer were included. The median age was 57 years (IQR: 50–64), and 73.7% were postmenopausal. The median BMI was 26.3 kg/m^2^ (IQR: 23.4–31.2), with 60.2% classified as overweight (BMI ≥ 25 kg/m^2^). Nulliparity was noted in 31.8%, and 53.3% had at least one comorbidity, most commonly hypertension (81.1%) and diabetes (47.3%). Advanced‐stage disease (FIGO III–IV) was present in 27.1%, high‐grade tumors in 30.5%, and non‐endometrioid histology in 18.3%. Median tumor size was 4.5 cm (IQR: 3–6.5), with LVSI present in 32.4%. After primary surgery, 54.6% received adjuvant therapy. Recurrence occurred in 9.3% and sites of recurrence was vaginal, peritoneum, lymph nodes, and distant were 2.9%, 2.1%, 2.7%, and 3.7%, respectively. Median SWT and time to adjuvant therapy were 32 days (IQR: 18–45) and 28 days (IQR: 22–42.2), respectively. Median follow‐up was 23 months (IQR: 11–49) (Table 1).

**TABLE 1:** Descriptive characteristics of endometrial cancer patients according to surgical waiting time (*n* = 377).

Variables	All patients (*n* = 377)	Surgical waiting time		*p*
		≤ 4 weeks (*n* = 164)	> 4 weeks (*n* = 213)	
Age (year), median (IQR)	57 (50,64)	58 (51.8,66.2)	57 (49,63)	0.057
Menopause, *n* (%)	278 (73.7)	126 (76.8)	152 (71.4)	0.232
BMI, median (IQR)	26.3 (23.4,31.2)	25.5 (22.9,29.5)	27.6 (24.2,32.4)	0.002*
BMI ≥ 25 kg/m^2^, *n* (%)	227 (60.2)	87 (53)	140 (65.7)	0.013*
Nulliparity, *n* (%)	120 (31.8)	56 (34.1)	64 (30)	0.397
Underlying disease, *n* (%)	201 (53.3)	79 (48.2)	122 (57.3)	0.079
Hypertension, *n* (%)	163 (81.1)	62 (78.5)	101 (82.8)	0.446
Diabetes mellitus, *n* (%)	95 (47.3)	31 (39.2)	64 (52.5)	0.067
Dyslipidemia, *n* (%)	89 (44.5)	35 (44.3)	54 (44.6)	0.964
Others, *n* (%)	32 (8.5)	13 (7.9)	19 (8.9)	0.732
FIGO stage III‐IV, *n* (%)	102 (27.1)	38 (23.2)	64 (30)	0.136
Non‐endometrioid, *n* (%)	69 (18.3)	25 (15.2)	44 (20.7)	0.178
Grade 3, *n* (%)	115 (30.5)	45 (27.4)	70 (32.9)	0.257
Size (cm), median (IQR)	4.5 (3,6.5)	4 (3,6)	5 (3.2,6.5)	0.038*
LVSI positive, *n* (%)	122 (32.4)	49 (29.9)	73 (34.3)	0.366
Adjuvant treatment, *n* (%)	206 (54.6)	90 (54.9)	116 (54.5)	0.936
T‐adj.(day), median (IQR)	28 (22,42.2)	27 (22,41)	28.5 (21.2,42.8)	0.930
Recurrence, *n* (%)	35 (9.3)	8 (4.9)	27 (12.7)	0.010*
Sites of recurrence				
Vaginal, *n* (%)	11 (2.9)	2 (1.2)	9 (4.2)	0.123
Peritoneum, *n* (%)	8 (2.1)	1 (0.6)	7 (3.3)	0.145
Lymph nodes, *n* (%)	10 (2.7)	4 (2.4)	6 (2.8)	1
Distant, *n* (%)	14 (3.7)	4 (2.4)	10 (4.7)	0.251

*Note:* Others underlying disease included asthma, hyperthyroid, stroke, chronic kidney disease (CKD) and heart disease such as valvular heart disease.

* indicates statistical significance (*p* < 0.05).

Abbreviations: BMI, body mass index; FIGO, International Federation of Gynecology and Obstetrics; IQR, interquartile range; LVSI, lympho‐vascular space invasion; Size, tumor size; T‐adj., time interval from surgery to adjuvant treatment.

Patients were stratified by SWT: ≤ 4 weeks (*n* = 164) versus > 4 weeks (*n* = 213). Those with delayed surgery had a significantly higher median BMI (27.6 vs. 25.5 kg/m^2^, *p* = 0.002), more frequent overweight status (65.7% vs. 53.0%, *p* = 0.013), and larger tumor size (median 5.0 vs. 4.0 cm, *p* = 0.038). Recurrence was significantly more frequent in the delayed group (12.7% vs. 4.9%, *p* = 0.010). No other significant clinicopathological differences were noted between the groups (Table 1).

As shown in Table 2, univariate Cox regression analysis identified several factors significantly associated with tumor recurrence, including FIGO stage III–IV (HR: 6.08, 95% CI: 2.98–12.39, *p* < 0.001), non‐endometrioid histology (HR: 2.42, 95% CI: 1.15–5.09, *p* = 0.020), grade 3 tumors (HR: 3.02, 95% CI: 1.52–5.98, *p* = 0.002), LVSI (HR: 4.14, 95% CI: 2.04–8.42, *p* < 0.001), and SWT > 4 weeks (HR: 3.20, 95% CI: 1.32–7.74, *p* = 0.010).

**TABLE 2:** Univariate and multivariate analyses of factors associated with tumor recurrence.

Variables	Tumor recurrence			
	Univariate analysis		Multivariate analysis	
	HR (95% CI)	*p*	HR (95% CI)	*p*
Age (year)	1.01 (0.98–1.05)	0.529	0.99 (0.95–1.03)	0.578
Menopause, yes versus no	0.84 (0.40–1.77)	0.654	0.55 (0.18–1.63)	0.281
BMI (kg/m^2^), ≥ 25 versus < 25	0.90 (0.45–1.80)	0.770	0.93 (0.46–1.87)	0.831
Parity, yes versus no	1.02 (0.48–2.14)	0.962	0.91 (0.40–2.09)	0.830
Underlying disease, yes versus no	1.17 (0.59–2.33)	0.647	1.26 (0.60–2.65)	0.541
FIGO stage, III–IV versus I–II	6.08 (2.98–12.39)	< 0.001*	5.15 (2.46–10.77)	< 0.001*
Non‐endometrioid versus endometrioid	2.42 (1.15–5.09)	0.020*	0.97 (0.37–2.55)	0.943
Grade, 3 versus 1–2	3.02 (1.52–5.98)	0.002*	2.19 (0.85–5.63)	0.103
Tumor size (cm), ≥ 2 versus < 2	1.30 (0.31–5.45)	0.716	1.15 (0.27–4.86)	0.853
LVSI, positive versus negative	4.14 (2.04–8.42)	< 0.001*	2.20 (0.99–4.93)	0.054
Surgical waiting time (SWT, weeks)				
> 4 versus ≤ 4	3.20 (1.32–7.74)	0.010*	3.22 (1.31–7.93)	0.011*
> 6 versus ≤ 6	2.34 (1.18–4.63)	0.015*	3.22 (1.52–6.81)	0.002*
> 8 versus ≤ 8	2.29 (1.11–4.72)	0.025*	3.16 (1.43–7.02)	0.005*

*Note:* Underlying disease included hypertension, diabetes mellitus, dyslipidemia, asthma, stroke, chronic kidney disease, hyperthyroid and heart disease. Multivariate analysis adjusted by Age, BMI, FIGO stage, grade and histology. SWT was analyzed using three different cutoff points (4, 6, and 8 weeks) to evaluate its impact across various clinically relevant thresholds, including the common practice in Thailand (4 weeks) and thresholds reported in international literature (6 and 8 weeks).

* indicates statistical significance (*p* < 0.05).

Abbreviations: BMI, body mass index; CI, confidence interval; FIGO, The International Federation of Gynecology and Obstetrics; HR, hazard ratio; LVSI, lympho‐vascular space invasion.

In the multivariate model adjusting for age, BMI, FIGO stage, grade, and histology, two factors remained independently associated with recurrence: advanced‐stage disease (HR: 5.15, 95% CI: 2.46–10.77, *p* < 0.001) and SWT > 4 weeks (HR: 3.22, 95% CI: 1.31–7.93, *p* = 0.011). Further analysis by extended SWT thresholds demonstrated a persistent and statistically significant increase in recurrence risk. Patients with SWT > 6 weeks (HR: 3.22, 95% CI: 1.52–6.81, *p* = 0.002) and > 8 weeks (HR: 3.16, 95% CI: 1.43–7.02, *p* = 0.005) remained at elevated risk compared to those undergoing surgery within 4 weeks. These findings underscore a consistent and dose‐dependent relationship between surgical delay and tumor recurrence (Table 2). SWT did not independently predict long‐term OS in this cohort; advanced stage remained the only independent predictor of both PFS and OS (*p* < 0.001) (Table 3).

**TABLE 3:** Univariate and multivariate analyses of factors associated with overall survival.

Variables	Overall survival			
	Univariate analysis		Multivariate analysis	
	HR (95% CI)	*p*	HR (95% CI)	*p*
Age (year)	1.05 (1.01–1.08)	0.015*	1.03 (0.99–1.07)	0.118
Menopause, yes vs. no	1.80 (0.75–4.35)	0.190	0.82 (0.26–2.59)	0.732
BMI (kg/m^2^), ≥ 25 vs. < 25	1.16 (0.58–2.31)	0.671	1.48 (0.74–2.94)	0.270
Parity, yes vs. no	1.22 (0.57–2.60)	0.610	0.83 (0.37–1.87)	0.657
Underlying disease, yes vs. no	1.85 (0.92–3.73)	0.084	1.69 (0.80–3.57)	0.171
FIGO stage, III–IV vs. I–II	7.52 (3.67–15.38)	< 0.001*	5.92 (2.80–12.50)	< 0.001*
Non‐endometrioid vs. Endometrioid	3.50 (1.78–6.88)	< 0.001*	1.15 (0.46–2.88)	0.757
Grade, 3 vs. 1–2	4.18 (2.13–8.23)	< 0.001*	2.18 (0.84–5.61)	0.107
Tumor size (cm), ≥ 2 vs. < 2	0.77 (0.23–2.53)	0.669	0.67 (0.19–2.29)	0.520
LVSI, positive vs. negative	3.70 (1.86–7.36)	< 0.001*	1.60 (0.74–3.43)	0.231
Surgical waiting time (SWT, weeks)				
> 4 vs. ≤ 4	1.87 (0.87–3.99)	0.107	1.99 (0.91–4.34)	0.083
> 6 vs. ≤ 6	1.19 (0.60–2.36)	0.623	1.87 (0.89–3.93)	0.099
> 8 vs. ≤ 8	1.04 (0.46–2.39)	0.919	1.92 (0.75–4.92)	0.172

*Note:* Underlying disease included hypertension, diabetes mellitus, dyslipidemia, asthma, stroke, chronic kidney disease, hyperthyroid and heart disease. Multivariate analysis adjusted by Age, BMI, FIGO stage, grade and histology. SWT was analyzed using three different cutoff points (4, 6 and 8 weeks) to evaluate its impact across various clinically relevant thresholds, including the common practice in Thailand (4 weeks) and thresholds reported in international literature (6 and 8 weeks).

* indicates statistical significance (*p* < 0.05).

Abbreviations: BMI, body mass index; CI, confidence interval; FIGO, The International Federation of Gynecology and Obstetrics; HR, hazard ratio; LVSI, lympho‐vascular space invasion.

**FIGURE 2:**
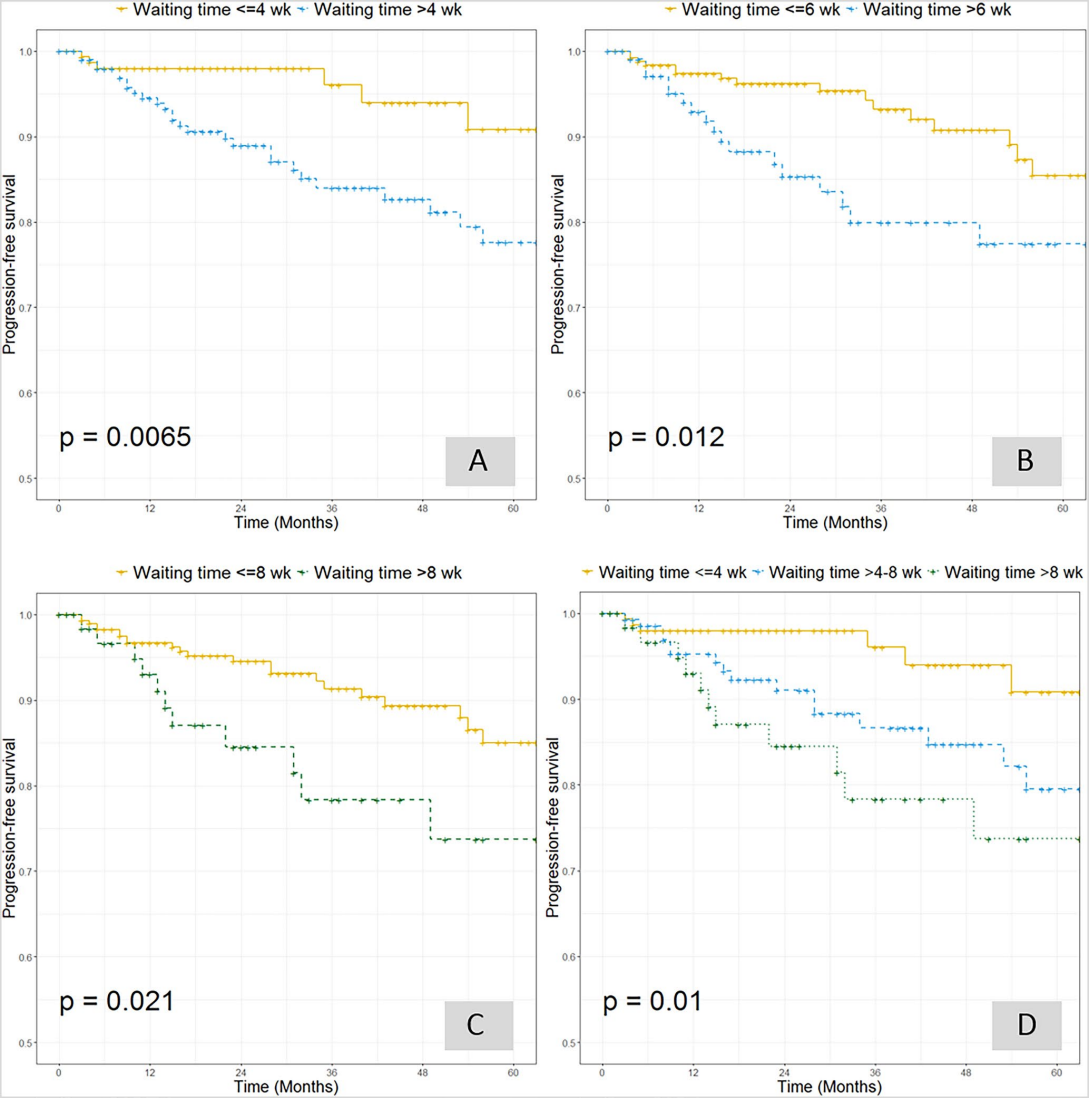
Progression‐free survival (PFS) according to surgical waiting time (SWT). Kaplan–Meier curves showing PFS stratified by different SWT thresholds. (A) PFS for patients with SWT ≤ 4 weeks versus > 4 weeks (*p* = 0.0065). (B) PFS for patients with SWT ≤ 6 weeks versus > 6 weeks (*p* = 0.012). (C) PFS for patients with SWT ≤ 8 weeks versus > 8 weeks (*p* = 0.021). (D) PFS for patients with SWT ≤ 4 weeks, 4–8 weeks, and > 8 weeks (*p* = 0.01). Curves indicate that longer surgical waiting times are associated with reduced PFS.

Kaplan–Meier analysis demonstrated that median PFS was 21 months (IQR: 10–46), with a 5‐year PFS rate of 82.8% (95% CI: 76.9–89.2). Patients who underwent surgery within 4 weeks had significantly better PFS (90.8%, 95% CI: 82.9–99.5) compared to those with SWT > 4 weeks (77.6%, 95% CI: 69.7–86.5) (*p* = 0.0065; Figure 2A). This trend persisted when using alternative cutoff points of 6 weeks (*p* = 0.012; Figure 2B) and 8 weeks (*p* = 0.021; Figure 2C). Subgroup analysis categorizing patients into three groups (≤ 4 weeks, > 4–8 weeks, > 8 weeks) revealed the poorest PFS among those with surgery delayed beyond 8 weeks (*p* = 0.01; Figure 2D).

**FIGURE 3:**
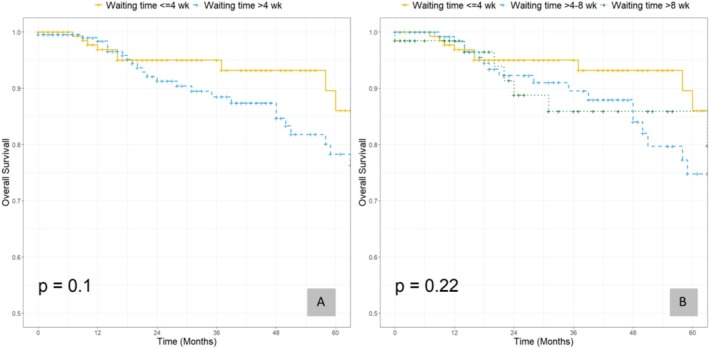
Kaplan–Meier analysis of overall survival (OS) according to surgical waiting time (SWT). (A) OS in patients stratified by SWT ≤ 4 weeks versus > 4 weeks. No significant difference in OS was observed between the two groups (*p* = 0.10). (B) OS in patients stratified by SWT ≤ 4 weeks, > 4–8 weeks, and > 8 weeks. Long‐term survival was comparable across groups, with no statistically significant difference (*p* = 0.22).

The median OS was 23 months (IQR: 11–50), with a 5‐year OS rate of 81.1% (95% CI: 74.6–88.2). Stratified by SWT, the 5‐year OS was 86.0% (95% CI: 76.0–97.4) for SWT ≤ 4 weeks and 78.3% (95% CI: 70.1–87.5) for SWT > 4 weeks. Kaplan–Meier analysis showed that SWT was not significantly associated with 5‐year OS compared with the 4‐week reference (*p* = 0.10) (Figure 3A) and demonstrated no significant difference among the three SWT groups (≤ 4 weeks, > 4–8 weeks, and > 8 weeks; *p* = 0.22) (Figure 3B).

## 4 Discussion

This study provides compelling evidence that prolonged SWT significantly impacts the prognosis of patients with EC. We found that longer SWT are directly associated with increased tumor recurrence and diminished 5‐year PFS. Our detailed analysis, examining SWT across clinically relevant intervals of 4, 6, and 8 weeks, offers crucial insights into the time‐dependent nature of oncologic risk. This clearly positions SWT as a modifiable determinant of outcome, underscoring the urgent need for timely surgical intervention in EC treatment.

Our results strongly align with Thailand's national Key Performance Indicator (KPI), which advocates for a 4‐weeks benchmark for cancer surgery. However, patients operated on within 4–8 weeks showed comparable 5‐year PFS to those treated within 4 weeks and better outcomes than those waiting > 8 weeks (*p* = 0.01). Regarding OS, no significant differences were observed between SWT ≤ 4 and > 4 weeks (*p* = 0.10) or among the three SWT groups (*p* = 0.22). Taken together, these findings suggest that surgery within 8 weeks remains acceptable in real‐world settings without compromising survival, particularly for patients with early‐stage or low‐risk disease.

These observations are consistent with previous studies linking treatment delays to worse outcomes in various cancers. Khorana et al. [4] reported that prolonged time to treatment initiation negatively impacted survival in breast, lung, renal, and pancreatic cancers. Similarly, in EC, studies by Shalowitz et al. [10], Strohl et al. [11], AlHilli et al. [12], and Pergialiotis et al. [13] found that delays beyond 6–8 weeks were associated with increased mortality or recurrence. Together, these findings further reinforce the need for timely surgical care.

However, not all studies report consistent findings. Marcickiewicz et al. [14] and Matsuo et al. [15] observed no survival disadvantage in patients undergoing surgery up to 70 days after diagnosis. Similarly, Plubprasit et al. [16] found no significant difference in 5‐year disease‐free survival when comparing SWT intervals of 4 and 6 weeks. Interestingly, subgroup analysis in that study showed that delays had a more pronounced impact among patients with advanced‐stage disease or non‐endometrioid histology—findings that align with our results. These inconsistencies may stem from differences in population characteristics, study design, or unmeasured confounding variables. While minimizing delays is crucial, it is also important to consider the concept of individualized surgical timing. Some studies [10, 14] have paradoxically linked excessively early surgery (within 15 days) to poorer outcomes, potentially due to selection bias towards patients with more aggressive disease or limited physiological reserve who might undergo expedited procedures.

From a biological standpoint, it is plausible that the observed adverse impact of prolonged SWT stems from tumor progression during delays, facilitated by mechanisms such as deeper myometrial invasion, angiogenesis, immune evasion, and epithelial‐mesenchymal transition—particularly in high‐grade or non‐endometrioid tumors [17, 18]. Similar trends have been observed in cervical cancer; Nanthamongkolkul and Hanprasertpong [5] reported that delays beyond 8 weeks were associated with significantly reduced survival, suggesting shared biological vulnerabilities across gynecologic malignancies.

The PFS curves illustrate the adverse impact of surgical delay, with patients undergoing surgery within 4 weeks showing over 95% PFS for nearly 3 years—likely reflecting early‐stage, low‐risk profiles. In contrast, delayed‐surgery patients more frequently had non‐endometrioid histology, larger tumors, and higher BMI, contributing to poorer outcomes. Several factors may explain this association. Obese patients often present with increased perioperative risks, necessitating additional preoperative assessments (e.g., cardiopulmonary evaluation, anesthetic consultation) and optimization of comorbidities such as diabetes or hypertension, which can delay scheduling. Similarly, larger tumors may prompt further imaging or multidisciplinary discussions to plan the optimal surgical approach or anticipate intraoperative challenges, thereby extending the waiting time. To reduce SWT in these groups, strategies could include establishing fast‐track preoperative pathways for high‐BMI patients, earlier referral to anesthesiology and internal medicine for risk optimization, and standardized protocols for evaluating large tumors to minimize unnecessary delays. Streamlined operating room scheduling and prioritization policies for high‐risk cases may also help ensure timely surgery without compromising safety. Although these findings are biologically plausible, they should be interpreted with caution, as patients who underwent delayed surgery must have survived the waiting period, which may have influenced survival estimates. Additionally, the small size of certain subgroups limits the robustness of stratified analysis.

Although 27.1% of our cohort had advanced‐stage (FIGO III–IV) and many had high‐grade tumors, the overall recurrence rate was unexpectedly low (9.3%). This may reflect the benefits of standardized multidisciplinary care in a tertiary referral center, where optimized surgical planning, appropriate use of adjuvant therapy, and structured follow‐up may collectively improve outcomes. In particular, multidisciplinary input likely contributed to better patient selection for surgery, timely initiation of adjuvant treatments, and comprehensive surveillance—all of which may have reduced recurrence risk despite high baseline pathology. In multivariate analysis, histologic subtype, tumor grade, and LVSI were no longer significant, likely due to overlap with advanced FIGO stage and delayed surgery. These findings suggest that overall disease burden and treatment timing may have a stronger prognostic impact than individual pathological factors. Another potential source of bias is the exclusion of higher‐risk patients (*n* = 134), such as those who received systemic treatment before surgery, were referred after inadvertent or incomplete surgery, or had concurrent malignancies or rare uterine sarcomas. This selection process may have led to an underestimation of recurrence rates, as these excluded patients could have harbored more aggressive disease or worse prognostic profiles.

Importantly, even early‐stage, low‐grade EC may be negatively impacted by delayed surgery. Nwachukwu et al. [19] reported that biopsy‐to‐surgery intervals ≥ 6 months were associated with markedly higher 3‐year recurrence rates (54% vs. 8%), with timing remaining an independent predictor. These results further support the need to maintain a 4‐week surgical window, regardless of initial risk classification.

Despite growing awareness, surgical delays remain common. O'Leary et al. [20] reported that over half of patients with uterine cancer underwent surgery beyond 6 weeks—a trend mirrored in our cohort, where 56.5% had SWT > 4 weeks. Addressing systemic barriers, including referral inefficiencies, preoperative preparation delays, and operating room availability, is essential for improving surgical timeliness, particularly among high‐risk populations.

The COVID‐19 pandemic has further exacerbated surgical delays. Singh et al. [21] described postponements of up to 8 weeks in gynecologic cancers, while Alkatout et al. [22] reported deferments of 10–12 weeks for low‐grade EC, with interim hormonal therapy as a bridging strategy. These disruptions underscore the need for healthcare systems to maintain cancer care continuity—even during crises—by balancing infection control with oncologic urgency.

Our results demonstrated that prolonged SWT impact on OS was less pronounced. Although delayed surgery may increase the risk of early recurrence, long‐term survival appears to be influenced primarily by subsequent interventions, such as salvage therapy for recurrent disease, with the site of recurrence potentially affecting survival outcomes. These observations align with prior studies [5, 14, 16], reinforcing the notion that OS is less sensitive to moderate surgical delays.

While current NCCN guidelines for EC do not specify a defined timeframe for surgical intervention, our results support the implementation of a 4‐week window post‐diagnosis. Incorporating timing benchmarks into clinical practice guidelines and national policy frameworks may enhance treatment consistency, reduce variability, and support quality assurance in gynecologic oncology.

This study has several limitations. First, its retrospective design introduces inherent selection bias and unmeasured confounders. Second, as a single‐center analysis, the findings may not be fully generalizable to other settings. Third, the relatively short median follow‐up of 23 months may have underestimated late recurrences or survival events, potentially influencing the observed association between SWT and PFS. Longer follow‐up could better clarify the long‐term oncologic impact of surgical delays. Finally, prospective multicenter studies with extended follow‐up are warranted to validate and expand these findings.

SWT beyond 4 weeks and advanced stage were independently associated with increased recurrence and inferior 5‐year PFS in patients with EC. Surgery within 4 weeks should be prioritized, and delays exceeding 8 weeks should be avoided, particularly in patients with advanced‐stage or high‐risk histologic subtypes. These findings support Thailand's KPI and underscore the need for timely surgery and system‐level efforts to reduce delays in EC management. However, the relatively short median follow‐up period (23 months) may limit the validity of these findings, especially regarding long‐term outcomes such as overall survival. Longer follow‐up is warranted to confirm these results.

## Author Contributions

**Ascharavadee Pulsawat:** conceptualization, investigation, writing – original draft, methodology, validation, visualization, writing – review and editing, formal analysis, data curation. **Sitchuphong Noothong:** conceptualization, writing – review and editing. **Nathapol Sirimusika:** writing – review and editing, investigation.

## Funding

The authors have nothing to report.

## Disclosure

The authors have nothing to report.

## Ethics Statement

This study was approved by the Ethics Committee of HatYai Hospital (Study code: HYH EC 044‐68‐01) and conducted in accordance with the Declaration of Helsinki and relevant local regulations.

## Consent

The authors have nothing to report.

## Conflicts of Interest

The authors declare no conflicts of interest.
